# Environmental Stress-Induced Alterations in Embryo Developmental Morphokinetics

**DOI:** 10.3390/jox14040087

**Published:** 2024-10-21

**Authors:** Dorit Kalo, Shira Yaacobi-Artzi, Shir Manovich, Ariel Michaelov, Alisa Komsky-Elbaz, Zvi Roth

**Affiliations:** Department of Animal Sciences, Robert H. Smith Faculty of Agriculture, Food and Environment, The Hebrew University, Rehovot 7610001, Israel; dorit.kalo@mail.huji.ac.il (D.K.);

**Keywords:** embryo morphokinetics, environmental stressors, time-lapse system

## Abstract

The association between embryo morphokinetics and its developmental competence is well documented. For instance, early cleaved embryos are more competent in developing to blastocysts, whereas the proportion of abnormally cleaved embryos that further developed to blastocysts is low. Numerous factors, such as the parental age, lifestyle, health, and smoking habits have been reported to affect the embryo morphokinetics and, consequently, its development. However, less is known about the effect of environmental stressors on embryo morphokinetics. The current review discusses the effect of the most concerning environmental stressors on embryo morphokinetics. These stresses include heat stress and human-made chemicals such as phthalates (e.g., bis-(2-ethylhexyl phthalate, dibutyl phthalate, dimethyl phthalate, and their primary metabolites), herbicides (e.g., diaminochlorotriazine, the primary metabolite of atrazine), pharmaceutical compounds (e.g., carbamazepine, nocodazole) and pro-oxidant agents (cumene hydroperoxide, Triton X-100), as well as naturally occurring toxins such as mycotoxin (e.g., aflatoxin B1 and its metabolite, and ochratoxin A). In addition, this review discusses the effect of ionizing or non-ionizing radiation and viral infections (e.g., SARS-CoV-2, papillomavirus). Finally, it points out some potential mechanisms that underlie the impairment of embryo morphokinetics, and it suggests protective compounds, mainly the supplementation of antioxidants to improve the morphokinetics, and consequently, the embryo developmental competence.

## 1. Embryo Morphokinetics

The developmental morphokinetics of the embryo is associated with its developmental competence. Time-lapse systems enable a larger and deeper view of the developmental competence of the preimplantation embryo and help explore its morphokinetics. The term morphokinetics includes the time interval between cell divisions combined with the morphological features of the embryos: (i) the time between fertilization and the appearance of the pronucleus [1], (ii) the appearance of nuclei after the first cleavage [2], (iii) the duration of the first cytokinesis [3,4], (iv) the time between the first and second mitosis [5,6], (v) the synchronicity of the second and third mitosis [3,4,7], and (vi) the expansion and hatching time of the blastocyst [8,9]. These parameters have been suggested as a tool for predicting embryo viability, its competence to implant, establishing pregnancy, and achieving a live birth [10,11]. 

**Figure 1:**
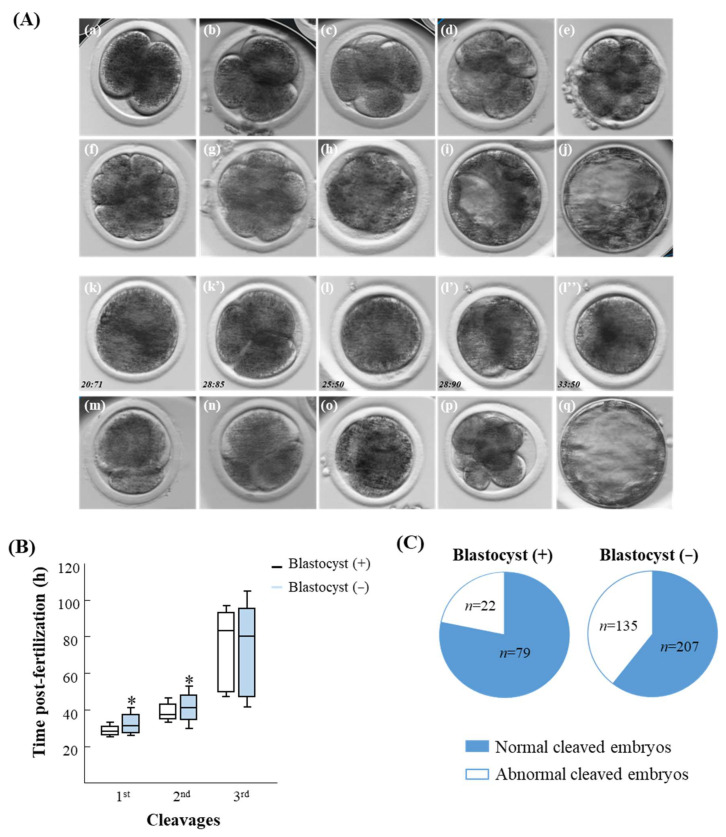
Embryo morphokinetics via the time-lapse system. (**A**) Representative images of a normally cleaved embryo through different developmental stages: (**a**) 2-cell, (**b**) 3-cell, (**c**) 4-cell, (**d**) 5-cell, (**e**) 6-cell, (**f**) 7-cell, (**g**) 8-cell, (**h**) morula, (**i**) an early blastocyst, and (**j**) an expanded blastocyst, and an abnormally cleaved embryo: (**k**,**k’**) a directly cleaved embryo from zygote to 3-cell, (**l**,**l’**,**l’’**) a reversed cleaved embryo from zygote to the 2-cell embryo and back to the 1-cell embryos, (**m**) a 2-cell stage embryo displaying unequally sized blastomeres, (**n**) a 2-cell stage embryo with large fragments, i.e., classified as having poor morphology, (**o**) a 4-cell stage embryo displaying unequal sized blastomeres, i.e., classified as having poor morphology, (**p**) an 8-cell stage embryo displaying unequally sized blastomeres, i.e., classified as having poor morphology, (**q**) a blastocyst exhibiting a poor morphological appearance, with loosely clustered cells at the inner cell mass and a trophoblast containing a layer of discontinuous cells. (**B**) Presented is the cleavage timing post-fertilization (1st, 2nd, and 3rd cleavages) of cleaved embryos that further developed (+) or did not (−) develop to the blastocyst stage [25]. The Kruskal–Wallis test, followed by the Wilcoxon test for pairwise comparisons, was used to compare the median time values of the 1st, 2nd, and 3rd divisions of embryos that either developed to blastocysts or did not. Data are presented in box and whisker plots, indicating the timing for 25, 50 (i.e., median), and 75% of the cleaved embryos. * *p* < 0.05. (**C**) Presented is the distribution of cleaved embryos classified as either with a normal or abnormal cleavage pattern among embryos that either developed (+; *n* = 101, the total number of embryos that developed to the blastocyst stage) or did not develop (−; *n* = 342, the total number of embryos that did not reach the blastocyst stage) to the blastocyst stage [25] . The chi-squared test, followed by Fisher’s exact test, was performed to compare the cleavage pattern distribution (normal or abnormal) of embryos that developed to blastocysts with those that did not.

Based on a time-lapse system, discrimination embryos can be classified into two developmental patterns: normal and abnormal. Embryos are classified as normal, when the cell cleaves into two equally sized blastomeres. The normally cleaved embryo can be further divided into two sub-categories: those embryos that represent synchronous (i.e., divisions into 4, 8, and 16 blastomeres) or asynchronous (i.e., divisions into 3, 5, 6, 7, 9, 10, or 12 blastomeres) cleavages (Figure 1A) [12,13]. The abnormal cleavages can be distinguished by one or more abnormalities. These cleavages include the direct cleavage when the zygote divides directly into >3 blastomeres [14], the unequal cleavage, when the zygote divides into two unequally sized blastomeres [15,16], and the reverse cleavage, when the number of blastomeres after division is reduced (Figure 1A) [5,17]. In addition, the time-lapse system allows embryo morphological classification at any developmental stage. Parameters such as the shape, color, number, and compactness of blastomeres, as well as the degree of fragmentation are used to classify the embryo as good, fair, or poor [12,15,18,19,20]. Taken together, examining embryo morphokinetics enables one to predict more accurately the developmental competence of the embryo. Numerous studies have indicated that embryos classified as normal and/or early divided in the first division have a higher probability to develop to the blastocyst stage [5,10,12,14,21,22,23]. More specifically, we reported that bovine embryos that developed to the blastocyst stage were faster in the 1st (i.e., division from 1-cell to 2-cell stage embryo) and 2nd (i.e., division from 2-cell to 4-cell stage embryo) divisions rather than those that did not develop to the blastocyst stage [5,12]. In addition, embryos displaying normal cleavage patterns exhibited higher chances to develop into blastocysts than did the abnormal ones [5,12]. Moreover, embryos that further developed into blastocysts displayed good morphology relative to the arrested ones [12,24]. In support, we recently encountered similar results using a new data set (Figure 1B,C; [25]). With respect to embryo morphology, previous studies indicated the usefulness of embryo scoring as a pregnancy predictor [26]. However, blastocysts that developed from normally and abnormally cleaved embryos displayed a similar morphology [12], which indicates that morphology should be considered with caution. Taken together, the establishment of a time-lapse imaging algorithm, mainly in IVF clinics, provides a superior tool for selecting embryos, rather than using the common subjective morphological evaluations. 

## 2. Potential Factors That Affect Embryo Morphokinetics

Several factors were found to affect embryo morphokinetics, for instance, parental age (maternal and/or paternal), smoking habits, spermatozoa quality, and obesity [27,28,29,30,31,32,33]. Assistance reproductive technology (ART) methods such as stimulation protocol, fertilization method (IVF vs. ICSI), and culture medium were also found to affect the developmental morphokinetics [2,33,34,35,36,37]. A delay in the time of the division into 2-, 4-, and 7-cell stage embryos was reported when fertilization was performed with compromised spermatozoa, i.e., lower concentration and motility [32]. In addition, the time from pronuclei fading to the 2-cell stage and from the 2- to 3-cell stage was found to be longer. Moreover, a higher proportion of abnormal division patterns, mainly direct cleavage and higher fragmentation incidences was reported [32]. Another study reported that the kinetics of the developing embryos differ between fertilization with fresh and surgically derived spermatozoa, mainly expressed by a slower division into the 8-cell stage and a delay in embryo compaction and blastulation [37]. Increased maternal, as well as paternal, age is also associated with delayed kinetics, mainly in the first three divisions [30,31,38,39]. On the other hand, the association between the incidence of abnormal cleavages and maternal age is controversial [39,40,41]. In contrast, increased paternal age was reported to be associated with a higher proportion of abnormal cleavages, such as direct and reverse [30]. With further insight into embryo culture conditions, besides the culture medium, the incubator environment, including the temperature, oxygen levels, and osmotic pressure, can potentially affect embryo morphokinetics [42]. Although much has been reported regarding the best conditions for human embryonic development (for a review, see [43]), less is known about the impact on embryo developmental morphokinetics. For instance, a low oxygen level (i.e., 5%) during embryo culture has been suggested in many animal studies, since it was highly associated with a better developmental outcome (for a review, see [44]). For human embryos, information regarding the proper oxygen level is not clear cut [44]. Whereas some reports indicate that altering the oxygen level during culture does not affect embryo morphology [45], other reports indicate that embryos exhibit lower fragmentation when cultured at 5% O_2_, compared with those cultured at 20% O_2_ [46]. The benefit of culturing at 5% oxygen was demonstrated regarding the timing of the third division, e.g., the embryos displayed faster division at the third division [36]. Another aspect of the culture condition that is associated with in vitro embryonic development is the media osmolality [47]. The media osmolality can be affected, for example, by the humidity in the incubator [48], which can affect embryo developmental morphokinetics. For example, embryos cultured under relatively high humidity conditions exhibit a faster pronucleus appearance and fading, the time of the first division, and the time of morula development [49]. 

## 3. Environmental Stressors

**Figure 2:**
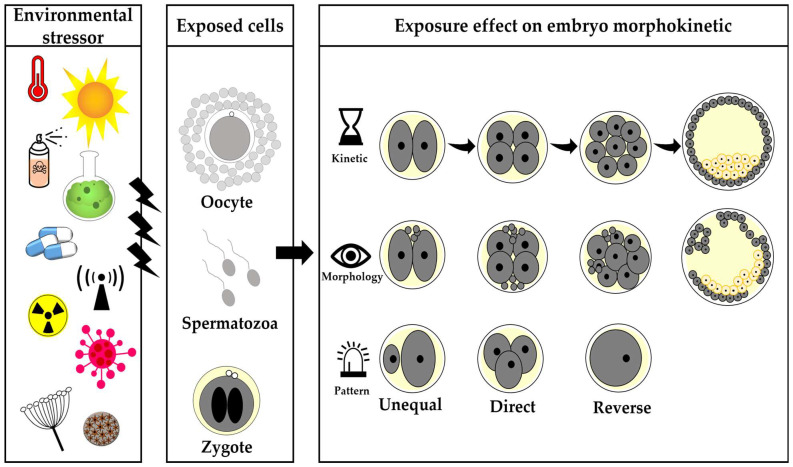
Schematic illustration of the most concerning environmental stressors that affect embryo morphokinetics. These stressors include heat stress as well as human-made chemical and naturally occurring compounds. They have been reported to affect the oocyte, spermatozoa, or zygote, which are further expressed by embryo kinetics, embryo morphology, and embryo division patterns.

Various environmental stressors were reported to affect the oocyte and/or the sperm, thereby impairing the developmental competence of the resulting embryo [50,51]. The current review discusses the effect of the most concerning environmental stressors on embryo morphokinetics. These stresses include heat stress, endocrine-disrupting compounds, mycotoxins, viruses, pharmaceutical compounds, and radiation (Figure 2). In addition, this review also specifies some potential protective compounds that might improve the embryo morphokinetics and its developmental competence.

### 3.1. Effect of Heat Stress on the Embryo Developmental Morphokinetics

Summer heat stress is a major environmental factor that contributes to low fertility in lactating dairy cows, manifested by immediate and long-lasting deleterious effects on the female reproductive system [52]. Seasonal studies in dairy cows reported that environmental heat stress negatively affects embryonic development. For instance, environmental heat stress was associated with an 8–12% embryonic loss throughout 21 to 30 days of gestation [53]. Exposing cows to thermal stress during the hot season reduces the proportion of oocytes that undergo fertilization and that developed to 2-cell, 8-cell stage embryos, morula, and blastocysts [54,55]. Similarly, exposing oocytes to elevated temperatures during in vitro maturation results in a decreased cleavage rate and a reduced proportion of oocytes that develop to the blastocyst stage [56,57,58]. Other reports indicate that not only the oocyte itself is sensitive to thermal stress—the formed embryos are also sensitive to it [52,59]. However, the association between thermal stress and embryo developmental morphokinetics has been studied less.

**Figure 3:**
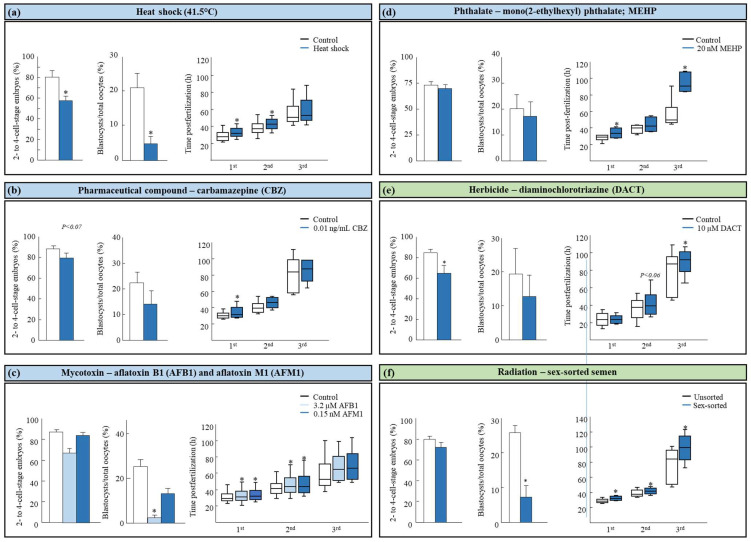
The effect of various environmental stressors on embryonic developmental morphokinetics. Presented are carry-over effects from the oocyte (in blue) or the spermatozoa (in green) to the developed embryo. Environmental stressors include (**a**) heat shock (i.e., 41.5 °C; modified from Yaacobi-Artzi et al. [61]), (**b**) 0.01 ng/mL of carbamazepine (CBZ; modified from Kalo et al. [70]), (**c**) 3.2 µM of aflatoxin B1 (AFB1) and 0.15 nM of AFM1 (modified from Yaacobi-Artzi et al. [71]), (**d**) 20 nM of mono (2-ethylhexyl) phthalate (MEHP; [72]), (**e**) 10 µM of diaminochlorotriazine (DACT; modified from Komsky-Elbaz et al. [73]), and (**f**) sex-sorting methodology (modified from Kalo et al. [74]). Embryo developmental competence was analyzed by one-way ANOVA, followed by Student’s *t*-test or by the Tukey–Kramer test; data are presented as the mean ± SEM. Embryo kinetics through the first three divisions were analyzed using the Kruskal–Wallis test, followed by the Wilcoxon test to compare the median time values of the 1st, 2nd, and 3rd divisions between groups. Data are presented in box and whisker plots, indicating the timing for 25, 50 (i.e., median), and 75% of the cleaved embryos * *p* < 0.05.

A recent seasonal study in which oocytes were collected during the hot season (June–September) or the cold season (December–May) found a seasonal variation in embryo developmental kinetics [60]. A delay in the second division was recorded post-fertilization for oocytes that were collected during the hot season. This delay was accompanied by reduced blastocyst formation. In support, Gendelman et al. [61] found a delay in the timing of the first cleavage for in vitro-derived embryos that developed from oocytes collected during the hot season relative to those collected during the cold season. The developmental peak to the 2-cell stage was higher and occurred about 13 h earlier in the cold season. A similar delay was found for the second division to the 4-cell stage, manifested by a higher proportion of 2-cell stage embryos versus 4-cell stage embryos at 42 h post-fertilization in the hot season [61]. With respect to embryo morphological scoring, interesting findings were found, indicating a higher proportion of good grade embryos during the hot season in the 1st, 2nd, and 3rd divisions [60]. In vitro exposure to a physiological thermal stress caused a delay in the first and the second divisions as well as a delayed time to blastocyst formation [62]. Early cleaved embryos are more likely to develop to the blastocyst stage than late-cleaving embryos [12,21,22] and are associated with higher pregnancy rates and lower abortion frequencies [63]. Therefore, heat stress-induced alteration in the embryo kinetics of the first divisions is suggested as one of the mechanisms that impair the embryo developmental competence (Figure 3). Alterations in the temperature gradient in in vitro culture systems were also found to impair mouse morphokinetics [64,65]. 

Heat stress can also affect the proportion of abnormally cleaved embryos, manifested by a higher proportion of unequally cleaved embryos in the hot season relative to the cold season (14.3 vs. 5.9%, respectively) [60]. Recent studies in ovine, bovine, and porcine found that the proportion of blastocysts that developed from unequally cleaved embryos was lower than those that developed from normally cleaved embryos [66,67,68]. Therefore, the heat that increased the proportion of abnormally cleaved embryos might partially explain the reduced embryonic development in bovine during the summer months. A recent seasonal study examined the morphokinetics of in vivo-derived bovine embryos using a time-lapse system [69]. The investigators used machine learning in which the pixel changes from one frame to the next, defined as the morphokinetic activity, and found a differential morphokinetic activity between seasons, manifested by increased metabolism. Such alterations were suggested to reduce the embryo competence to establish pregnancy. 

### 3.2. Effect of Human-Made Chemicals on the Embryo Developmental Morphokinetics

Various compounds in the environment were found to have a deleterious effect on embryo developmental competence. Some of them are human-made chemicals, whereas others are naturally occurring toxins. About 80,000 compounds, regardless of their source, have been defined as endocrine disruptor compounds (EDCs) due to their ability to mimic, block, or interfere with the endogenous hormone cascade. Given the extreme number of existing compounds, the current review discusses only some representative compounds that are associated with reproductive health, in particular, those compounds that affect embryo developmental morphokinetics.

Phthalates: The phthalate group contains more than 25 types that are used for commercial applications. The bis-(2-ethylhexyl phthalate) (DEHP) is the most commonly used phthalate [75] and can be found in toys, vinyl flooring, wall covering, detergents, lubricating oils, food packaging, pharmaceuticals, blood bags, and personal care products [76,77]. Farm animals can be exposed to phthalate via milking equipment, such as milking pipes, liners, teat dip cups, food packing, and silage wrap [78]. In the body, DEHP is metabolized into several metabolites; the major metabolite is the mono(2-ethyhexyl) phthalate (MEHP), which has higher toxic effects than DEHP [76,79]. Phthalates were detected in female follicular fluid; they are associated with a negative reproduction outcome [80,81]. Similarly, Phthalates were found in the follicular fluid of lactating cows associated with reduced oocyte developmental competence [82]. Moreover, blastocysts that developed from 20 nM of MEHP-treated oocytes expressed a different transcriptomic profile [83]. 

An association between phthalate exposure and embryo morphokinetics was reported. Exposing equine oocytes to 500 nM DEHP during in vitro maturation affects the morphokinetics of the developing embryos, manifested by a lengthy time from fertilization to the extrusion of the second polar body and a shorter cell cycle time from the 2- to 3-cell stage [80]. In addition, a higher proportion of embryos expressed an abnormal division pattern, such as a direct first cleavage in the DEHP-treated group [84]. In agreement, we recently found that exposing bovine oocytes to 20 nM of MEHP results in a 5 h delay from fertilization to the first division into the 2-cell stage, relative to the untreated oocyte (33.7 ± 2.5 vs. 28.1 ± 0.9%, respectively, *p* = 0.04). A delay in the third division was also demonstrated (Figure 3; [72]). Studies in mice found that exposing 2-cell stage embryos to 10^−3^ M of monomethyl phthalate (MMP), a dimethyl phthalate metabolite, slows the developmental rate into the 4-, 8-cell stage, and to the morula and the blastocyst stages. Slowing the cleavage rate was also associated with increased reactive oxygen species (ROS) and decreased ATP synthesis [85]. On the other hand, exposure to 10^−3^ M of mono-n-butyl phthalate (MBP), a dibutyl phthalate (DBP) metabolite, did not impair the kinetics of mouse embryos into the 2-, 4- and 8-cell stages, but it reduced the blastocyst formation rate [86]. Exposing the oocytes of *Galeolaria caespitose*, a marine invertebrate, to 0.2, 2, and 20 mg/mL of DBP did not impair the proportion of embryos with normal vs. abnormal morphology. Interestingly, fertilization with DBP-treated *G. caespitosa* spermatozoa resulted in a high proportion of embryos with an abnormal morphology at the 4-, 8-, and 16-cell stage, in a dose-dependent manner [87], suggesting a paternal effect on embryo developmental morphokinetics. 

Other synthetic compounds: Many chemical and synthetic compounds were suggested to negatively affect embryonic development. These compounds include chemicals such as parabens, bisphenols, microplastic, triclosan, per- and poly-fluoroalkyl substances (PFASs), perfluorinated compounds, and more [88]. Bisphenol A (BPA), for example, is the most studied bisphenol [89]. In vitro maturation of bovine oocytes with Bisphenol A (BPA) reduces both the cleavage and the blastocyst formation rate [90,91]. Moreover, BPA also impairs the expression of some miRNA in the oocytes [90], as well as the blastocyst metabolism [91]. However, the association between embryo development and morphokinetics is less known. One study on mouse oocytes indicated that BPA delays the cell cycle through meiosis [92], suggesting a possible impact on the embryo morphokinetics following fertilization. On the other hand, the BPA concentration in body fluids (urine, plasma, and follicular fluid) did not affect the IVF outcome, expressed as the proportion of good quality embryos or the proportion of normally fertilized oocytes [93]. Whereas some studies suggest a negative correlation between PFASs in women’s follicular fluid and the oocyte fertilization rate or the IVF outcome [94,95], other studies reported that there is no association [96,97]. In vitro maturation of bovine oocytes with perfluoroalkyl acids, a subgroup of PFASs, reduced the cleavage and blastocyst formation rates, but it did not alter the blastocyst morphology [98]. Taken together, although PFASs were reported to have a negative impact on the female reproductive system (for a review, see [99]), and to some extent on embryonic development, the effect of PFASs on the embryo morphokinetics remains obscure. 

Herbicides and pesticides: The increasing use of herbicides and pesticides in agriculture has become, in turn, a global health problem [100,101,102]. About 1000 of these compounds were identified as EDCs [103]. Some of these compounds were reported to affect proper embryonic development in livestock [104]. For instance, atrazine is a chlorotriazine herbicide [105], which is known as a ubiquitous environmental contaminant [106] and is considered an EDC [107,108]. A study in bovine reported that atrazine and its primary metabolite diaminochlorotriazine (DACT) induces a carry-over effect, from the sperm to the embryo [73]. Pre-fertilization exposure of fresh spermatozoa to 10 µM of DACT impairs the morphokinetics of early cleaved embryos, manifested by a delay in the division time into the 4- and 8-cell stages (Figure 3). This delay was associated with a higher incidence of abnormal cleavage patterns, mostly a reverse cleavage. Other commonly used herbicides that were reported to affect embryonic development are BASTA-15^®^ and ROUNDUP^®^. In vitro exposure of bovine semen to Glyphosate, the active ingredient in ROUNDUP^®^ herbicide [109], did not impair the spermatozoa’s ability to fertilize oocytes, expressed by the cleavage rate; however, it reduced the proportion of embryos that developed to blastocysts [110]. Moreover, the addition of ROUNDUP^®^ herbicide during oocyte maturation reduced both the cleavage and blastocyst formation rate [110]. In addition, the in vitro maturation of porcine oocyte with Glyphosate did not impair oocyte nuclear maturation or cleavage rate but it did reduce the proportion of developed blastocysts [111]. Similarly, the in vivo administration of BASTA-15^®^ to dams reduced the proportion of morulas and early blastocysts, and the majority of the embryos arrested at earlier cleavage stages (up to 16 cells) [112]. In addition, the in vitro culturing of mouse embryos with BASTA-15^®^ negatively affected embryo growth and quality even at low concentrations [112]. Currently, the effect of these compounds on the embryo morphokinetics is less known. 

Pharmaceutical compounds: The presence of pharmaceutical compounds in the environment is a world-wide concern [113]. Several pharmaceutical compounds were found in the soils and in crops that were irrigated with treated waste water [114,115]. These compounds include carbamazepine, lamotrigine, caffeine, metoprolol, sulfamethoxazole, sildenafil, and others [115]. Among these compounds, carbamazepine, an antiepileptic drug, is one of the most frequently detected pharmaceuticals in the environment [116,117]. Another study in male rats showed that carbamazepine impairs steroidogenesis and sperm quality, decreases testosterone levels, and delays puberty [118]. Administration of carbamazepine during pregnancy decreases fetal weight, decreases the crown-rump distance [119,120], and increases the proportion of pre- and post-implantation embryonic loss [119]. Carbamazepine also impairs the embryo morphokinetics; we recently found that exposing the oocytes during maturation to carbamazepine at 0.01 mg/mL causes a delay in the division timing into the 2-, 4- and 6-cell stage, and exposure to 0.1 ng/mL induces a delay to the 6- and 7-cell stage embryos. In addition, a higher proportion of abnormally cleaved embryos was recorded in the treated group following exposure to 0.01 or 0.1 ng/mL of carbamazepine (*p* = 0.02) [70] (Figure 3). In mice, maternal exposure to carbamazepine from day 9 of gestation induced a delay in the developmental kinetics of embryos [121]. 

Another sample of pharmaceutical compound found in treated wastewater is the nocodazole, an antifungal drug [122]. It is known to disrupt the microtubule organizational capability, which in turn, leads to spindle abnormalities and chromosome segregation errors [123,124,125]. A recent study in mice found that nocodazole affects the morphokinetics of oocytes during in vitro maturation. In particular, it affects the timing to the first polar extrusion, which was longer, resulting in a prolonged meiosis I [125]. 

Oxidation agents: Oxidation agents are intensively used to induce oxidative stress in many biological models [126,127]. For instance, Cumene hydroperoxide was reported to induce oxidative stress, expressed by lipid peroxidation and DNA damage [128]. Similarly, Triton X-100, a human-made chemical, is mainly used in biochemical applications to induce oxidative stress [129,130]. Both chemicals (i.e., Cumene hydroperoxide and Triton X-100) were reported to affect the embryo morphokinetics. Culturing cryopreserved 1-cell mouse embryos with either Cumene hydroperoxide or Triton X-100 delays the timing of divisions and the cell cycle length [131]. For instance, cumene hydroperoxide at concentrations of 4, 6, and 8 µM induced a delay in the division timing into the 4-, 5-, 6-, 7-, and 8-cell stage, as well as in the time of morula formation, blastocoel cavity formation, and blastocyst expansion. Similarly, exposure to a low level (0.0004%) of Triton X-100 had a prominent effect on the timing of the blastocoel cavity formation, manifested by a delay of ~5 h [131]. Note that the timings of morula compaction and blastocyst formation are both markers for blastocysts of high quality. A rapid morula compaction and faster blastocyst formation were found to be associated with greater chances to develop to the blastocyst stage, establish pregnancy, and achieve live births [132,133]. 

Mineral oil is routinely used to protect the in vitro-cultured embryos from inadequate medium temperature, pH, and osmolality [134]. The mineral oil is composed of crude oil and contains unsaturated hydrocarbons, peroxides, and Triton-X 100. The two latter ones are known as embryo-toxic elements, which, under inappropriate storage conditions, can form free radicals [134,135]. A study conducted in mice reported that clinical-grade mineral oil impairs the embryo morphokinetics, manifested by an increased duration of the cell cycle and a delay in the division into the 2- and 8-cell stage, and the formation time of the blastocyst [136]. Wolff et al. [131] reported that using recalled mineral oil for mouse embryo culture caused a delay in division into the 3-, 4-, 5-, 6-, 7-, and 8-cell stages, as well as morula and blastocyst formation. Although not clear enough, the mineral oil seems to have a toxic effect on embryo morphokinetics, presumably via the induction of oxidative stress by ROS and other free radial agents; thus, it should be handled with caution. In support of this assumption, a study conducted in porcine oocytes reported an increase in the oxidant status of the culture medium following the use of Peroxidized mineral oil, i.e., an altered batch of mineral oil [137].

Taken together, human-made chemicals seem to have deleterious effects on the embryo developmental morphokinetics (Table 1), consequently impairing the proportion of embryos that develop into blastocysts. One of the suggested mechanisms underlying this impairment, is elevation in ROS levels above the required physiological level during preimplantation [138]. This point requires further investigation. 

### 3.3. Effect of Natural Occurring Compounds on the Embryo Developmental Morphokinetics

Naturally occurring compounds are derived from biological sources, such as mycotoxins, which are produced by fungi, chemicals from plant or soil origin, and physical source components, such as metal, glass, and gas (e.g., radon). In the current review, we aimed to discuss representative compounds, mainly mycotoxin, and their effect on embryo morphokinetics. Although not classified as toxins, we will also address the effect of viruses on the embryo morphokinetics, since viruses, for instance, SARS-CoV-2, naturally exist in the environment [139].

Mycotoxins: Aflatoxins are naturally occurring toxins that belong to a large group of mycotoxins produced by the fungi *Aspergillus flavus* and *Aspergillus parasiticus* [140]. Aflatoxins can be found in food products, grains, fruits, nuts, and other crops, especially under humid conditions [141]. Among the aflatoxins, aflatoxin B1 (AFB1) is highly toxic to mammals, due to its carcinogenic, mutagenic, and teratogenic effects [142,143]. In the body, AFB1 is catabolized into various metabolites; the main ones are AFM1 and AFQ1 [144]. AFM1 is classified as carcinogenic and immunosuppressive and can be found in the milk of animals that were fed contaminated feedstuff [145]. Accordingly, permissible limits for AFB1 and AFM1 concentrations in food were established for humans (0.01–0.1 μM) and animals (up to 1 μM) [144,146]. 

Aflatoxins are considered risk factors for infertility [147]. Accumulating studies indicated that mammalian oocytes are vulnerable to aflatoxins, in particular, to AFB1. Studies in porcine [148], ovine [149], bovine [71], and mice [150] found that AFB1 affects meiosis and might involve alterations in the cell cycle mechanism. In addition, exposing mouse oocytes to AFB1 impairs the endoplasmic reticulum, the Golgi, and the mitochondrial distribution [150]. Exposing ovine oocytes during maturation to AFB1 reduces their mitochondrial membrane potential in association with elevated ROS [149]. Exposing rat oocytes to AFB1 results in a reduced fertilization rate [151] and a decrease in the cleavage and blastocyst formation rates [149]. Similarly, culturing bovine embryos for 7.5 days post-fertilization with AFB1 reduces the cleavage and blastocyst formation rates [152]. 

Aflatoxins also affect embryo morphokinetics. A recent study in bovine reported that AFB1 increases the proportion of asynchronously cleaved embryos [71]. Both AFB1 and AFM1 induce alterations in the kinetics of cleaved embryos in a dose-dependent manner, manifested by a delay in the first, second, and the third divisions [71] (Figure 3). Given that the three first embryo divisions are associated with embryo developmental competence, aflatoxins are suggested to affect further stages of embryo development. Both AFB1 and AFM1 alter embryo morphology. For instance, AFB1 decreased the number of embryos displaying fair morphology in the 2nd division (i.e., moderately irregular blastomeres in shape, size, or color (at least 50% intact) [71]. In addition, AFM1 altered embryo morphology in the 2nd and 3rd divisions, as well as blastocyst morphology in a dose- dependent manner [71]. In accordance, a recent study in sheep examined the effect of Ochratoxin A, a natural mycotoxin produced by several fungi of *Aspergillus* and *Penicillium* [153]. Exposing ovine oocytes to Ochratoxin A during maturation increases the rate of embryos that arrested at the 2–4-cell and 8–16-cell stages, in a dose-dependent manner [153]. In addition, treatment with Orchatoxin A induces a delay in the division time into the 5-cell and 8-cell stages, with no difference at other time points of division. Note that both aflatoxin and Ochratoxin A do not affect the embryo morphology, indicating that embryo morphology is not a good parameter by itself to assess the embryo quality [13]. 

Viral infection—a lesson from the SARS-CoV-2 and papillomavirus: The outbreak of corona disease (COVID-19) has been a high concern of the World Health Organization (WHO). COVID-19 is a highly transmittable and pathogenic viral infection caused by the severe acute respiratory syndrome coronavirus 2 (SARS-CoV-2) [154]. SARS-CoV-2 invades the target host cells via binding to the angiotensin-converting enzyme 2 (ACE2) membrane receptor [155]. Human oocytes and embryos express the ACE2 receptor [156,157], which make them vulnerable to SARS-CoV-2 infection. A recent work reported that SARS-CoV-2-infected patients that undergo IVF procedures have a lower number of retrieved oocytes [158]. However, the maturation, fertilization, and blastocyst formation rates did not differ from those of uninfected patients [158]. Nonetheless, the number of 3-day embryos that were of high quality and suitable for transfer was lower in the SARS-CoV-2-infected patients. With respect to the embryo developmental kinetics, no differences were recorded in the time from fertilization to pronuclei fading and the time from fertilization to division into 2-, 3-, 4-, or 5-cell embryos. On the other hand, a delay of 1–2 h was found at each developmental stage from the 6-cell stage until early blastulation in the SARS-CoV-2-infected group; this action was accompanied by a prolonged duration from the 2- to 3-cell stage [158]. In support, Braga et al. [159] reported that the kinetics of cleaved embryos were delayed in the SARS-CoV-2-infected group compared with healthy counterparts. This delay included a longer time to pronuclei appearance, time to pronuclei fading, time to 2-, 3-, 4-, 5- cells, and time to blastulation. The duration of the transition time from the pronuclei fading to the 2-cell, 3- to 5-cell stage, and from the 3- to 4-cell stage increased in the infected group as well. Nevertheless, SARS-CoV-2 infection did not affect the implantation and pregnancy rates [159,160,161]. Although not clear enough, it is suggested that alterations in the embryo morphokinetics could result from SARS-CoV-2 interference with mitotic and cytoskeleton arrangement during embryonic divisions. This assumption is based on the understanding that, following invasion into the host cells, SARS-CoV-2 cooperates with the cytoskeletal network, i.e., the microtubules, actin, and the microtubule organization [162]. 

The papillomavirus, a DNA virus, is also reported to alter the embryo morphokinetics. Human embryos from positively infected patients showed faster kinetics in the early stages. This was manifested by a shorter pronuclei fading time following IVF or ICSI procedures; a shorter interval was recorded between the appearance and fading of the pronuclei, resulting in earlier division to the 2-cell stage. Nonetheless, the time of blastulation was found to be slower in the virus-infected group compared with the non-infected one [163]. It was suggested that papillomavirus affects the spindle assembly checkpoint, which in turn, leads to mitotic defects [164].

Taken together, the findings clearly indicate that virus infections, SARS-CoV-2, and papillomavirus are associated with morphokinetic alterations through meiosis and/or embryonic development. 

Findings indicate that not only human-made chemicals impair the embryo developmental morphokinetics, but natural occurring compounds as well (Table 2). Based on this understanding, further research should be performed in this field to clarify the effects of other chemicals and/or numerous compounds in the environment.

### 3.4. Effect of Radiation on the Embryo Developmental Morphokinetics

In general, radiation is classified as either ionizing or non-ionizing. There are three main types of ionizing radiation: the alpha, beta, and gamma rays (or the X-ray). Owing to intensive technological progress, exposure to ionizing radiation is unavoidable. The ionizing radiation is characterized by high energy that can directly affect the DNA structure [171,172], or it can induce the generation of ROS and oxidative stress [173]. Ionizing radiation during pregnancy, mainly preimplantation, was reported to deleteriously affect the embryo [174,175]. On the other hand, Jacquet [176] stated that exposing mice to a very low dose of ionizing radiation did not impair the morphology and the transcription in preimplantation embryos. Moreover, exposing women to controlled X-rays during hysterosalpingography results in a beneficial effect on the oocyte developmental competence, reflected by a higher fertilization rate, higher blastocyst formation, and higher proportion of blastocysts of good quality [177]. 

A recent study reported that exposing bovine ovaries to X-ray radiation (100 mGy) affects the developmental morphokinetics of the embryos. The irradiated-cleaving embryos were divided into two groups: those that further developed to the blastocyst stage and those that did not [165]. It was found that the duration of the three first embryonic divisions was longer in the subgroup of embryos that did not develop to blastocysts. Exposing oocytes to X-rays increases the duration of the first division from the 2- to 3-cell stages and from the 3- to 4-cell stages but does not affect blastocyst formation. 

Exposure to electromagnetic radiation through a variety of electrical devices is a risk factor for reproductive health [178,179]. Nevertheless, to date, the reports are controversial; some reports indicate no significant effect on the fertilization rate and on early embryonic development [180,181], whereas others documented a reduction in the blastocyst formation rate and an increase in embryonic death [182]. Electromagnetic radiation was reported to affect the mitochondria and to induce oxidative stress [183]; exposing mouse zygotes to electromagnetic radiation (900–1800 Hz) increases the proportion of necrotic embryos at the 2-cell stage; no impact was recorded on the proportion of embryos that developed to blastocysts; however, blastocysts that developed from irradiated zygotes were less viable and expressed a higher cell death [181].

In recent studies, mouse zygotes were irradiated with electromagnetic radiation (900–1800 Hz) and further cultured in an incubator that was equipped with a time-lapse system [166,167]. The results indicated a reduction in blastocyst formation in association with kinetic impairments: in particular, a delay in the first divisions into the 2-cell stage to the 12-cell stage [166,167]. In addition, a delay in the kinetics was recorded in embryos that developed from irradiated zygotes; this delay included the time of blastocoel formation, the formation time to the blastocyst stages, and the time to hatching [167]. Electromagnetic radiation also affects the embryo morphology and the cleavage pattern. For instance, a higher proportion of abnormal divisions was obtained following radiation, mainly a reverse cleavage pattern, which is known to be associated with a higher proportion of embryos of low quality [166,167]. 

#### The Impact of Radiation on the Spermatozoa in the Embryo Developmental Morphokinetics

It is well documented that ionizing radiation has a negative impact on human spermatozoa, manifested by a reduced spermatozoa concentration and motility, impairment of spermatozoa morphology, induction of oxidative stress, and DNA damage [184]. A negative impact of UV lasers, such as DNA breakage, was reported in human spermatozoa [185]. Similarly, in vitro exposure of human spermatozoa to electromagnetic radiation results in decreased spermatozoa motility and viability as well as an increase in ROS levels [186]. A study in bovine found that in vitro fertilization of oocytes with UV-irradiated spermatozoa affects the fertilization capability of the spermatozoa, reflected by a reduction in the cleavage rate into the 2-cell stage at 48 h post-fertilization, along with the complete inhibition of blastocyst development [187]. 

In bovine, sorting semen is a widely used procedure to achieve the desired sex of offspring [188,189]. One of the sorting methodologies is based on the DNA difference between X- and Y-spermatozoa, which, in domestic animals, is about 4.5 to 3%, respectively [190]. The sorting process involves several intervention points that can potentially compromise the function and quality of the sperm [189]. The spermatozoa undergo staining and then are sorted via a flow cytometer in which the spermatozoa are exposed to the high pressure (40–50 psi) of UV-laser light [191,192]. It is worth mentioning that during the spermatozoa sorting procedure, the seminal plasma is removed; therefore, the fluid components, including antioxidants, are eliminated [193]. Taken together, the spermatozoa are not only exposed to radiation but are also prone to the harmful effect of oxidative stress [193]. Studies in stallion, in which spermatozoa were sorted by flow cytometry, reported a significant increase in the ROS level, DNA fragmentation, a decrease in the mitochondrial membrane potential, as well as reduced spermatozoa viability [193,194,195]. 

Nevertheless, although the effect on the spermatozoa has been well studied, the subsequent effect on the developing embryo is less known. A recent study from our laboratory revealed that in vitro fertilization with bovine semen results in faster embryo divisions throughout all the developmental stages for unsorted, relative to Y- or X-sorted, semen (Figure 3) [74]. It is worth mentioning that the blastocyst formation rate was higher in the unsorted relative to the sorted group. No differences were found between normally vs. abnormally cleaved embryos following fertilization with sex-sorted semen. In addition, in vitro fertilization with Y- or X-sorted semen did not alter the morphology of the developing embryos as well. In contrast, another study in bovine reported that in vitro fertilization with X-sorted semen results in a higher proportion of abnormally cleaved embryos, mainly reverse cleavage, and slower kinetics of the division into the 2-cell stage and to the morula stage. A delay in the blastocoel formation was also recorded [168]. Bermejo-Alvarez et al. [169] recorded a first cleavage delay following in vitro fertilization with sex-sorted semen in bovine. Steele et al. [170] reported no differences in the kinetic development of bovine embryos following fertilization with sex-sorted semen; however, an increase in unfertilized oocytes, an increase in arrested zygotes and arrested embryos at the 4-cell stage were demonstrated. The carry-over effect of the sorted semen on the embryo morphokinetics is not clear enough and requires further investigation. 

## 4. Potential Protective Compounds to Preserve the Embryo Morphokinetics

The mechanism underlying the effect of environmental stressors on embryo developmental morphokinetics is still an open question. Accumulating findings suggest that the mechanism involves the activation of oxidative stress and impairment of the anti-oxidation defense machinery. Over the time course of embryonic development, the ROS level and the antioxidant machinery are at a delicate balance [196]. Moreover, the oxidative status of the embryo correlates with the ability of the embryo to develop to a blastocyst of good quality [197]. Although low levels of ROS are required for normal embryonic development, high levels of ROS can lead to lipid peroxidation and can induce damage to the cell membrane as well as cause DNA fragmentation [196,198]. Consequently, the use of several antioxidant agents was suggested to minimize the impact of oxidative stress [198]. Some of these potential “protective” agents are discussed below. 

Melatonin: Melatonin is a natural antioxidant compound [199]. Previous studies in mice, porcine, goat, bovine, and humans reported the beneficial effects of melatonin on oocyte and embryonic development [200]. Nevertheless, the impact of melatonin is controversial and differs between studies in a dose-dependent manner. Although some doses of melatonin result in a negative impact on embryonic development [201], others do not affect it [202,203]. On the other hand, melatonin was reported to protect cells from radiation [204,205] and to improve, to some extent, the formation rate of blastocysts exposed to heat shock [201]. Melatonin increased the cleavage and blastocyst rates following the exposure of bovine oocytes to high oxygen conditions (21% *v*/*v*) during maturation [206]. In another study in bovine, supplementing melatonin to the maturation did not affect the morphokinetics of embryos that developed from heat-shocked oocytes; a similar division time into the 2-, 4-, 8-cell stages, and blastocyst formation were recorded for both melatonin-treated and control groups [62]. In addition, the proportion of embryos with abnormal patterns, mainly direct cleavage, did not differ between the melatonin-treated and untreated groups. On the other hand, melatonin improved the cryotolerance of bovine blastocysts that underwent vitrification, manifested by a higher hatching rate at 24–72 h after thawing [206].

Insulin-like growth factor-I (IGF-I): IGF-I is secreted by the oviduct [207], the uterus [208], and the embryo [209]; it possesses antioxidation capabilities. A study in bovine showed that IGF-I can eliminate the effects of heat shock on bovine embryos, manifested by reducing apoptosis and improving the competence to develop to the blastocyst stage [210]. IGF-I was found to reduce the effects of hydrogen peroxide in mouse preimplantation embryos [211]. In our lab, we found that the addition of IGF-I to the maturation medium did not affect the cleavage rate post-fertilization, but tended to increase the blastocyst formation rate. With respect to embryo morphokinetics, IGF-I did not affect the proportion of normally vs. abnormally cleaved embryos but increased the proportion of asynchronously cleaved embryos. Treatment with IGF-I increased the proportion of unequally cleaved embryos and reduced the proportion of directly cleaved embryos. In addition, the time from fertilization to the first division to the 2-cell stage, as well as the time to blastocyst formation was faster upon IGF-I administration. In addition, treatment with IGF-I increased the blastocyst recovery after cryopreservation of heat-shocked embryos [212]. 

Vitamins: Vitamins are organic compounds found in food and are highly important for controlling health and metabolism. Most of the vitamins participate indirectly or directly against oxidative stress and serve as biological antioxidants [213]. A recent work in humans suggests that the content of vitamins (A, E, D, and K) in the follicular fluid serves as a good marker for oocyte developmental competence [214]. Supplementation of vitamin A to the buffalo maturation medium increased the maturation rate and balanced the expression of antioxidant-related genes [215]. The addition of vitamin C to the in vitro maturation medium of bovine oocytes decreased ROS levels and increased the blastocyst yield [216]. A study in humans associated the concentration of several vitamins (A, E, D, and B6) in the follicular fluid with embryo morphokinetics [217]. A positive correlation was found between the levels of vitamin A and vitamin B6 in the follicular fluid and the division timing into the 5-cell stage embryo and the transition timing from the 2- to 3-cell stage. Moreover, a positive correlation was noted between embryo morphology and the presence of vitamins A and B6 in the follicular fluid. On the other hand, a negative correlation was found between the levels of vitamin D and vitamin E regarding the optimal kinetic timing from the 2- to 3-cell stage and from the 3- to 4-cell stages, respectively [217]. Taken together, it seems that an optimal range of vitamins levels within the follicular fluid might improve oocyte developmental competence. 

Other antioxidants: Acetyl-L-carnitine (ALC), N-acetyl-L-cysteine (NAC), and α-lipoic acid (ALA) were shown to have beneficial effects on embryos that developed under high-oxygen conditions. For instance, a study in bovine reported that the addition of NAC to the maturation medium increased the proportion of 2-cell stage embryos of good quality [218]. A study in buffalo reported that the addition of ALC to the culture media improved oocyte developmental competence and the cryotolerance of the developed blastocysts [219]. An impact on the embryo morphokinetics was also demonstrated; culturing mouse embryos with a mixture of ALC, NAC, and ALA and under an environment of high oxygen (20%) resulted in faster embryonic progression, manifested by earlier division to the 5-, 6-, 7- and 8-cell stage, faster morula compaction, and faster blastocyst formation and expansion [220]. In support, the treatment of aged mouse oocytes prior to fertilization with ‘Auraptene’, which possesses antioxidative properties, improved the morphokinetics of the developing embryos; the morphokinetic parameters were comparable to those of embryos that developed from young oocytes [221]. A retrospective study in humans compared women that consume Sinopol^®^, a mixture of Myo-Inositol, ALA, and folic acid vs. those women that consume only folic acid. The comparison indicated that a higher proportion of embryos displayed an optimal cleavage timing for the 2- and 3-cell stage in the ‘Sinopol’ group. The latter was associated with a significantly higher clinical pregnancy rate and a higher proportion of live births relative to the ‘folic acid’ group [222]. Another study in humans reported a positive effect of the ‘Mediterranean diet’, enriched with omega-3, vitamin D, and olive oil, on the embryo developmental morphokinetics. This positive effect was reflected by a shorter time of division from the 2-cell stage into the 4-cell stage and from the 5- to 8-cell stage [223]. Omega-3 was suggested as an antioxidant [224]; however, in vitro fertilization with spermatozoa collected from bulls that were fed omega-3-enriched food did not affect the developmental kinetics nor the proportion of normally vs. abnormally cleaved embryos [225]. 

Collectively, this evidence strongly suggests that embryo morphokinetics can be affected by supplementing antioxidant compounds to the diet or to the culture medium, consequently preventing some extent oxidative stress. 

## 5. Synopsis

Utilizing the time-lapse system in IVF clinics and for research purposes indicated that embryo morphokinetics through early developmental stages were associated with its ability to further develop to a blastocyst. The current review combines some of the prominent environmental stressors and specifies their effects on embryo developmental morphokinetics. Interestingly, this review again raised an old but unsolved question: “whether different stressors share a common mechanism”. The data discussed here indicate that various stressors induce oxidative stress downstream of the cascade, manifested by an increased ROS level, DNA fragmentation, and an impaired mitochondrial function. Sometimes, pretreatments with antioxidants alleviate, to some extent, the deleterious effects on embryo developmental morphokinetics. The authors believe that further transcriptomic, proteomic, and metabolomic studies are required to better understand the intracellular and molecular mechanisms that underlie these alterations. This knowledge might lead to developing new strategies to cope with this phenomenon.

## Figures and Tables

**Table 1:** Collective data associating morphokinetic alterations following exposure to human-made chemicals.

Stressor	Subtype Stressor	Species	Cell Type	The Effect	Reference
Phthalates	DEHP	Equine	Oocyte	Delay in the extrusion of the second polar body; reduced duration of the second cell cycle; increased abnormal divisions	Marzano et al. [84]
	MMP	Mice	2-cell stage embryo	Delay in the divisions into the 4- and 8-cell stages; delay in the morula and blastocyst formation	Tian et al. [85]
	MBP	Mice *Galeolaria caespitose*	2-cell stage embryo Oocyte Spermatozoa	No effect on abnormal morphology Increased abnormal morphology	Chu et al. [86]; Lu et al. [87]
	DBP				
Herbicides		Bovine	Spermatozoa	Delay in the divisions into 4- and 8-cells; increase in abnormal division pattern	Komsky-Elbaz et al. [73]
Pharmaceutical compound	CBZ	Bovine	Oocyte	Delay in the division into the 2- 4-, 6-, and 7-cell stages; increased abnormal divisions	Kalo et al. [70]
	Nocodazole	Mice	Oocyte	Delay in the time of the first polar body extrusion	Zhu et al. [125]
Oxidant agent	Cumene hydroperoxide	Mice	Oocyte	Delay in the divisions into the 4-, 5-, 6-, 7-, and 8-cell stages; delay in morula, blastocoel cavity formations, and blastocyst expansion	Wolff et al. [131]
	Triton-X 100	Mice	Oocyte	Delay in the divisions into the 2-, 3-, 4-, 5-, 6-, 7-, and 8-cell stages, morula formation, blastocoel cavity formation, and blastocyst expansion	Wolff et al. [131]
	Mineral oil (peroxidated)	Mice	1-cell embryo	Delay in the division into the 2-, 3-, 4-, 5-, 6-, 7-, and 8-cell stages; delay in morula and blastocyst formation	Wolff et al. [131]; Ainsworth et al. [136]

**Table 2:** Collective data associating morphokinetic alterations following exposure to natural occurring compounds.

Stressor	Subtype Stressor	Species	Cell Type	The Effect	Reference
Mycotoxins	AFB1, AFM1	Bovine	Oocyte	Delay in the first, second, and third divisions, affecting the ratio between synchronous vs. asynchronous cleavages	Yaacobi-Artzi et al. [71]
	Orchatoxin A	Sheep	Oocyte	Delay in the divisions into the 5- and 8-cell stages; embryonic arrest at the 2–4-cell and 8–16-cell stages	Dell’Aquila et al. [153]
Virus	SARS-CoV-2	Human	Positively infected women	Delay in the pronuclei appearance and in pronuclei fading; delay in the division into the 2-, 3-, 4-, and 5-cell stages; delay in blastocyst formation; prolonged transition time from the 2- to 3-cell, 3- to 4-cell, 3- to 5-cell stages, and from the 6-cell stage to blastulation	Ma et al. [158]; Braga et al. [159]
	Papillomavirus	Human	Positively infected women	A shorter pronuclei appearance and fading; faster division into the 2-cell stage; delay in blastocyst formation	Zullo et al. [163]
Radiation	X-ray	Bovine	Oocyte	Delay in the divisions from the 2- to 3-cell stages and from the 3- to 4-cell stages	Vazirov et al. [165]
	Electromagnetic	Mice	Zygote	Delay in the division into the 2-cell to the 12-cell stage; delay in blastocyst formation and hatching; increased abnormal divisions	Koohestanidehaghi et al. [166]; Seify et al. [167]
	UV-laser light (used in sex-sorting method)	Bovine	Spermatozoa	Delay in all embryonic divisions; increased abnormal divisions	Kalo et al. [74]; Magata et al. [168]
				Delay in the first division	Bermejo-Alvarez et al. [169]
				Arrest at the zygote and the 4-cell stages; no difference in embryo kinetics	Steele et al. [170]

## Data Availability

The raw data supporting the conclusions of this article will be made available by the authors on request.
